# Quantitative multi-slice spiral CT perfusion parameters as predictors of collateral status and 90-day functional outcome in acute ischemic stroke

**DOI:** 10.3389/fneur.2026.1858384

**Published:** 2026-07-06

**Authors:** Dan Zhu, Xiaozhou Ma, Yingzhi Jiao, Shuai Liu, Jinzhu Yan, Lixin Zhang

**Affiliations:** Imaging Center, Qianwei Hospital of Jilin Province, Changchun, Jilin, China

**Keywords:** 90-day functional outcome, acute ischemic stroke, collateral circulation, CT perfusion, hypoperfusion intensity ratio, modified Rankin scale

## Abstract

**Objective:**

To evaluate the utility of quantitative computed tomography perfusion parameters for assessing collateral circulation and predicting 90-day functional outcomes in acute ischemic stroke.

**Methods:**

This retrospective study included 82 patients who underwent perfusion imaging within 24 h of symptom onset. Parameters including relative cerebral blood flow, relative cerebral blood volume, mean transit time, time to maximum, and hypoperfusion intensity ratio were analyzed. Collateral status was classified using multiphase angiography, and 90-day outcomes were assessed using the modified Rankin Scale.

**Results:**

Patients with robust collateral circulation showed higher relative cerebral blood flow and volume and shorter perfusion times compared with those with poor collaterals. Hypoperfusion intensity ratio demonstrated strong diagnostic performance (area under the curve 0.925). For predicting unfavorable 90-day outcome, HIR achieved an AUC of 0.912 and showed greater discrimination than mismatch ratio in the present cohort, while the combined HIR–rCBF–Tmax model achieved an AUC of 0.938. Hypoperfusion intensity ratio correlated positively with functional disability, while relative cerebral blood flow correlated negatively with infarct volume. Favorable outcomes were more frequent in patients with robust collaterals.

**Conclusion:**

Quantitative CTP parameters bridge a natomical collateral assessment and downstream tissue-level perfusion. HIR may provide a particularly informative functional marker of collateral efficiency and 90-day prognosis beyond conventional mismatch assessment.

## 1 Introduction

Cerebrovascular emergencies are dominated by acute ischemic stroke (AIS)—a leading global driver of mortality and long-term disability (1). Compounded by an aging demographic and surging rates of metabolic risk factors like hyperlipidemia, diabetes mellitus, and hypertension, the AIS burden across China is accelerating rapidly (2). Following a large vessel occlusion, the viability of the downstream cerebral architecture hinges on a complex interplay of physiological variables. Chief among these is the collateral circulation. This ancillary vascular network serves a vital salvage function, sustaining residual tissue perfusion and blunting the expansion of the ischemic insult (3, 4).

Functioning primarily via the circle of Willis and leptomeningeal anastomoses, collateral vessels reroute blood to the starved ischemic territory (5). Decades of clinical data confirm a clear physiological advantage. Patients equipped with robust collateral flow experience suppressed infarct core expansion, minimized final lesion volumes, and a diminished propensity for hemorrhagic transformation following reperfusion therapy (6, 7). Consequently, characterizing this collateral reserve is no longer an academic exercise but a cornerstone of emergency AIS management. It is particularly crucial for identifying viable candidates for endovascular thrombectomy operating outside standard therapeutic time windows (8).

Over the past decade, multi-slice spiral computed tomography perfusion (CTP) has revolutionized the acute neuroimaging landscape. By tracking the first-pass dynamics of an iodinated contrast bolus, CTP generates critical parametric maps—specifically cerebral blood flow (CBF), cerebral blood volume (CBV), mean transit time (MTT), and the time to maximum of the tissue residue function (Tmax) (9, 10). These capillary-level hemodynamic blueprints separate irreversibly infarcted core from salvageable penumbra, directly informing reperfusion strategies. A complementary tissue-level marker derived from CTP is the hypoperfusion intensity ratio (HIR), calculated as the volume of tissue with Tmax >10 s divided by the volume with Tmax >6 s. Unlike CTA collateral grading, which provides a predominantly anatomical and macrovascular depiction of arterial filling, HIR characterizes the functional severity of perfusion delay within the downstream ischemic tissue (11, 12). It therefore reflects not merely whether collateral vessels are visible, but how effectively the available collateral pathways sustain tissue-level blood delivery (13, 14).

Despite the proven utility of these parameters, clinical literature remains heavily skewed toward patients with large vessel occlusions undergoing thrombectomy. What remains underexplored is the comprehensive application of multiple CTP variables to simultaneously gauge collateral competence and forecast short-term trajectories across a broader, more heterogeneous AIS population (15, 16). Furthermore, the ideal multi-metric combination for clinical prognostication is still the subject of debate. Accordingly, the principal aim of this study was not simply to reconfirm that favorable collaterals are associated with better perfusion. Rather, we sought to quantify the transition from macrovascular anatomical collateral filling on multiphase CTA to downstream tissue-level functional perfusion on CTP and to determine whether HIR and related perfusion biomarkers retained prognostic value after adjustment for treatment modality, recanalization status, stroke etiology, and major clinical covariates. We further compared HIR with the conventional mismatch ratio to evaluate which tissue-level construct more accurately represented 90-day functional prognosis. Ultimately, we aim to deliver robust, evidence-based imaging tools that seamlessly integrate into acute clinical decision-making.

## 2 Methods

### 2.1 Study design and cohort assembly

This was a study undertaken in compliance with the Declaration of Helsinki. We orchestrated a retrospective observational analysis within the Department of Neurology at Qianwei Hospital of Jilin Province, surveying cases managed between March 2024 and December 2025. Institutional Review Board and Ethics Committee authorization was secured. Given the retrospective architecture of the study, the mandate for written informed consent was waived. All protocols strictly adhered to the Declaration of Helsinki and institutional human subjects guidelines.

Candidate profiles were systematically isolated using our institutional Picture Archiving and Communication System (PACS) and electronic medical records. Individuals were included if they met the following criteria: (1) aged ≥ 18 years; (2) clinical AIS diagnosis, corroborated by neuroimaging and neurologic evaluation within 24 h of the last known well time; (3) CTA-confirmed stenosis or occlusion involving the intracranial internal carotid artery (ICA), the M1 segment of the middle cerebral artery (MCA), or the proximal M2 segment of the MCA; (4) execution of a standardized multi-slice spiral CTP protocol within 24 h of onset; and (5) comprehensive clinical documentation, incorporating both admission National Institutes of Health Stroke Scale (NIHSS) scores and 90-day mRS follow-up data.

To ensure cohort homogeneity, rigorous exclusion parameters were applied. We excluded patients exhibiting: (1) bilateral hemispheric lesions or posterior circulation strokes; (2) a baseline mRS > 1 indicating pre-existing neurological deficits from prior strokes; (3) non-contrast CT (NCCT) evidence of intracranial hemorrhage or primary hemorrhagic stroke; (4) technically deficient CTP acquisitions compromised by motion artifact; (5) documented iodine allergies or profound renal impairment (eGFR < 30 mL/min/1.73 m^2^); (6) concurrent life-threatening systemic emergencies, including acute myocardial infarction or advanced heart failure; and (7) absent 90-day follow-up metrics.

Our methodological framework necessitated the exclusion of posterior and bilateral strokes. The collateral grading metrics and relative perfusion baseline ratios utilized in this study are explicitly calibrated for unilateral anterior-circulation ischemia. Introducing bilateral pathology would irreparably compromise the integrity of contralateral reference measurements. Only patients with intracranial ICA, MCA-M1, or proximal MCA-M2 lesions were included. The M1 segment was defined as the MCA trunk extending from its origin to the primary bifurcation or trifurcation, whereas proximal M2 occlusion was defined as occlusion of a major division within the proximal sylvian fissure. Distal M2, M3, and M4 occlusions were not included because of their different perfusion territories and generally smaller ischemic burden. Through this rigorous consecutive screening pipeline, an analytical cohort of 82 eligible patients was finalized.

### 2.2 Neuroimaging acquisition protocol

In the emergency department, all participants were evaluated using a 256-slice multi-detector scanner (Revolution CT, GE Healthcare, Milwaukee, WI, USA). The tri-phasic imaging suite incorporated an initial NCCT, followed by CTP, and concluding with head and neck CTA. From table positioning to protocol completion, the entire sequence demanded roughly 15 min.

To map the CTP hemodynamics, we employed a shuttle mode technique providing 80 mm of total Z-axis coverage. This envelope captured the complete bilateral MCA territories, extending through the thalamus, basal ganglia, and supraganglionic regions. Scanning parameters dictated a tube voltage of 80 kVp, auto-modulated tube current of 150 mA, 0.5-s rotation speed, and 5 mm slice thickness spanning a 50-s total acquisition time. Contrast delivery involved a 50 mL bolus of non-ionic Iopamidol (370 mgI/mL, Bracco Diagnostics) injected at 5 mL/s through an 18-gauge antecubital line using a Medrad Stellant dual-head injector. A 30 mL saline chaser followed immediately at the same rate. Image capture commenced 5 s post-injection, generating 26 consecutive temporal frames to thoroughly document the contrast bolus first-pass dynamics.

Without delay, the CTA was acquired utilizing the remaining systemic contrast. Coverage swept from the aortic arch through the vertex at a 0.625 mm slice thickness and 0.984 pitch (120 kVp, automatic exposure control). A standard bolus-tracking threshold (100 Hounsfield units in the ascending aorta) governed the arterial phase timing.

### 2.3 Perfusion post-processing and data extraction

Raw CTP datasets were processed on a dedicated workstation using CT Perfusion 4D software (GE Healthcare), which provides semiautomated generation of perfusion maps and volumetric measurements based on a delay-insensitive singular value decomposition algorithm. The arterial input function was automatically selected from the contralateral MCA or anterior cerebral artery, and the venous output function was assigned to the superior sagittal sinus. All automatically generated arterial and venous curves, motion-correction results, perfusion maps, and lesion masks were independently reviewed by two neuroradiologists who were blinded to collateral grades and clinical outcomes. Manual correction was permitted only when automatic vessel selection, motion artifact, noise, or curve truncation produced an anatomically or physiologically implausible result. Disagreements were resolved by consensus or, when necessary, by a senior neuroradiologist.

Five core quantitative metrics were tabulated for every subject: (1) rCBF (mL/100 g/min), indexing affected hemisphere flow against the healthy contralateral mirror region; (2) rCBV (mL/100 g), denoting relative tissue blood volume; (3) MTT (seconds), capturing the temporal average of blood transit; (4) Tmax (seconds), marking the residue function peak delay relative to the AIF; and (5) HIR, mathematically derived by dividing the tissue volume exhibiting Tmax > 10s by the volume exceeding Tmax > 6 s (17).

Further volumetric parameters were defined. Ischemic core was classified as tissue registering rCBF < 30% of the normal contralateral hemisphere. The total hypoperfused tissue volume was defined as the volume with Tmax > 6 s. Ischemic penumbra was calculated as the Tmax > 6 s volume minus the ischemic core volume. The perfusion mismatch ratio was prespecified as the total hypoperfused tissue volume with Tmax >6 s divided by the ischemic core volume defined by relative CBF < 30% of the contralateral hemisphere. Thus, the numerator represented the total hypoperfused territory rather than the penumbra volume alone. Ischemic penumbra was calculated separately as the Tmax >6 s volume minus the ischemic core volume. For patients with an ischemic core volume of 0 mL, the mismatch ratio was mathematically undefined because of a zero denominator. These values were therefore coded as missing and excluded only from analyses involving mismatch ratio; the patients remained included in all other clinical, imaging, and outcome analyses. In the present cohort, two patients had an ischemic core volume of 0 mL, leaving 80 patients available for mismatch-ratio analyses.

Although fully automated platforms such as RapidAI are widely used in contemporary multicenter stroke trials, CT Perfusion 4D was the routinely available perfusion-processing platform at our institution during the study period. To minimize operator-dependent variability, we implemented a standardized semiautomated workflow, prespecified perfusion thresholds, blinded dual-reader review, and senior adjudication. Therefore, manual intervention was restricted to quality-control correction rather than subjective alteration of perfusion thresholds or outcome-oriented lesion delineation.

### 2.4 Multiphase CTA collateral stratification

Collateral architecture was quantified utilizing mCTA source data reconstructed across three distinct temporal windows: peak arterial, peak venous, and delayed venous phases. Scoring relied on Menon et al.’s six-tier mCTA grading system (18). Under this paradigm, a score of 0 signifies a catastrophic absence of collateral filling. Conversely, a score of 5 reflects robust, complete filling perfectly synchronized with the contralateral hemisphere. Working independently and blinded to both clinical endpoints and perfusion data, two neuroradiologists assigned these scores. Inter-rater reliability was validated via Cohen’s kappa coefficient.

This methodology stratified the cohort into two functional categories. The good collateral circulation group (mCTA 3–5) featured at least partial reconstitution with no more than a single-phase delay in the periphery. The poor collateral circulation group (mCTA 0–2) was characterized by minimal or absent collateralization, accompanied by profound peripheral filling delays or total territory drop-out (19). This specific dichotomous threshold is a well-established standard, previously cross-validated against digital subtraction angiography (20).

### 2.5 Clinical data and outcome tracking

Comprehensive baseline parameters were extracted from the institutional medical archive. Recorded data included demographic characteristics (age, sex, and BMI), vascular risk factors and comorbidities (atrial fibrillation, diabetes mellitus, hypertension, hyperlipidemia, smoking status, and coronary artery disease), and acute stroke characteristics, including admission NIHSS score, symptom-to-imaging interval, lesion laterality, and the location and segment of the target arterial lesion. Lesion laterality was categorized as left- or right-hemispheric involvement according to the symptomatic vascular territory identified on CTA and CTP. Laboratory and treatment modalities (conservative, intravenous thrombolysis, or mechanical thrombectomy) were also documented. For patients undergoing EVT, final angiographic reperfusion was graded according to the modified Thrombolysis in Cerebral Infarction scale, and successful reperfusion was defined as mTICI 2b–3. Two independent reviewers calculated the Alberta Stroke Program Early CT Score (ASPECTS) from the initial NCCT to quantify early ischemic tissue shifts.

Functional independence at 90 days, tracked via the mRS, served as the primary clinical endpoint. Trained stroke nurses administered these assessments through standardized outpatient exams or telephone interviews. We classified mRS scores of 0–2 as favorable outcomes (functional independence). Scores of 3–6 denoted unfavorable trajectories (severe disability or death). Secondary tracking monitored in-hospital mortality, radiographic hemorrhagic transformation within 7 days, and early neurological deterioration (a ≥ 4-point NIHSS spike within 72 h).

Stroke etiology was classified according to the TOAST criteria as large-artery atherosclerosis, cardioembolism, small-vessel occlusion, other determined etiology, or undetermined etiology. Classification was based on vascular imaging, electrocardiographic and cardiac monitoring findings, echocardiography, laboratory testing, and the available clinical records. Two stroke neurologists independently assigned the TOAST subtype, with disagreements resolved by consensus.

### 2.6 Statistical architecture

All statistical analyses were performed using MedCalc version 20.0 (MedCalc Software, Ostend, Belgium) and SPSS version 26.0 (IBM Corp., Armonk, NY, USA). Normality was assessed using the Shapiro–Wilk test. Continuous variables are presented as mean ± standard deviation or median with interquartile range, as appropriate, and categorical variables as frequencies and percentages. Between-group comparisons were performed using the independent-samples t test, Mann–Whitney U test, chi-square test, or Fisher’s exact test, according to data distribution and variable type.

Spearman rank correlation analysis was used to evaluate associations between 90-day modified Rankin Scale scores and CTP parameters. Univariable binary logistic regression was initially performed to identify variables associated with unfavorable 90-day functional outcome. Variables with *p* < 0.10 in univariable analysis, together with clinically relevant confounders, were considered for multivariable analysis. To reduce overfitting given the 44 unfavorable outcome events, the primary model was restricted to a parsimonious set of predictors, including HIR, admission NIHSS score, ischemic core volume, intravenous thrombolysis, endovascular thrombectomy, successful reperfusion, and TOAST classification. Because atrial fibrillation was closely related to the cardioembolic TOAST subtype, these variables were not included simultaneously; TOAST classification was retained in the primary model, whereas atrial fibrillation was assessed in a sensitivity analysis.

Multicollinearity was evaluated using variance inflation factors, with values >5 considered indicative of potentially important collinearity. Highly correlated perfusion variables, including HIR, Tmax, mismatch ratio, and ischemic core-related measurements, were not entered simultaneously into the same model. HIR was analyzed per 0.1-unit increase. Firth penalized logistic regression was performed as a sensitivity analysis to assess the robustness of the primary findings. Model calibration was evaluated using the Hosmer–Lemeshow goodness-of-fit test.

A separate multivariable logistic regression model was constructed to identify factors independently associated with poor collateral status. Adjusted odds ratios and 95% confidence intervals were reported.

Receiver operating characteristic curve analysis was performed to evaluate the ability of individual CTP parameters to identify poor collateral status and predict unfavorable 90-day functional outcome. Optimal cutoff values, sensitivity, and specificity were determined using the Youden index. For outcome prediction, the AUCs of HIR and mismatch ratio were compared using the DeLong test for correlated ROC curves. All tests were two-sided, and *p* < 0.05 was considered statistically significant.

## 3 Results

### 3.1 Baseline demographic and clinical topography

Our stringent screening protocol isolated 82 eligible AIS patients. Utilizing the mCTA collateral criteria, 58.5% (*n* = 48) of the cohort fell into the robust collateral category, while the remaining 41.5% (*n* = 34) demonstrated poor collateral flow. Diagnostic consensus between the evaluating neuroradiologists was remarkably high (*κ* = 0.82, 95% CI: 0.72–0.92).

Baseline age, sex, BMI, hypertension, diabetes mellitus, hyperlipidemia, smoking status, and lesion laterality were comparable between the two collateral groups (all *p* > 0.05). Left-hemisphere stroke occurred in 27 of 48 patients (56.3%) with robust collaterals and in 20 of 34 patients (58.8%) with poor collaterals (*p* = 0.817). The distribution of target arterial lesions, including intracranial ICA, MCA-M1, and proximal MCA-M2 lesions, also did not differ significantly between the groups (all *p* > 0.05). However, a distinct clinical profile emerged in the poor collateral group. These patients were disproportionately afflicted by atrial fibrillation (38.2% vs. 16.7%, *p* = 0.028). Furthermore, they presented with far more profound initial neurological deficits (median NIHSS 15 vs. 8, *p* < 0.001) and significantly depressed initial ASPECTS scores (median 6 vs. 8, *p* < 0.001). Interestingly, the critical onset-to-imaging temporal window was nearly identical across both groups (*p* = 0.352) (Table 1).

**Table 1:** Cohort demographics and clinical baselines segmented by collateral viability.

Clinical variable	Robust collaterals (*n* = 48)	Poor collaterals (*n* = 34)	Test statistic (t/χ^2^/Z)	*p* value
Age (years)	64.8 ± 11.3	67.2 ± 10.8	0.96	0.341
Male, *n* (%)	28 (58.3)	22 (64.7)	0.34	0.562
BMI (kg/m^2^)	24.6 ± 3.2	25.1 ± 3.5	0.67	0.505
Hypertension, *n* (%)	32 (66.7)	25 (73.5)	0.44	0.507
Diabetes mellitus, *n* (%)	14 (29.2)	13 (38.2)	0.74	0.389
Atrial fibrillation, *n* (%)	8 (16.7)	13 (38.2)	4.84	0.028*
Hyperlipidemia, *n* (%)	18 (37.5)	15 (44.1)	0.36	0.55
Current/past smoker, *n* (%)	20 (41.7)	16 (47.1)	0.23	0.631
Admission NIHSS, median (IQR)	8 (5–12)	15 (11–19)	−4.52	<0.001*
Initial ASPECTS, median (IQR)	8 (7–9)	6 (5–7)	−3.85	<0.001*
Onset-to-CT interval (min)	185 ± 95	205 ± 110	0.89	0.375
Target vessel occlusion, *n* (%)				
ICA	12 (25.0)	14 (41.2)	2.38	0.123
MCA-M1 segment	26 (54.2)	16 (47.1)	0.4	0.528
MCA-M2 segment	10 (20.8)	4 (11.8)	1.16	0.282
IVT, *n* (%)	18 (37.5)	10 (29.4)	0.62	0.431
EVT, *n* (%)	30 (62.5)	20 (58.8)	0.1	0.752
Successful recanalization among EVT-treated patients, n/N (%)	28/30 (93.3)	12/20 (60.0)	–	0.009*
TOAST – LAA, *n* (%)	18 (37.5)	8 (23.5)	1.98	0.16
TOAST – CE, *n* (%)	12 (25.0)	15 (44.1)	4.24	0.039*
TOAST – SVO, *n* (%)	10 (20.8)	3 (8.8)	2.45	0.118
TOAST – Other/Undetermined, *n* (%)	8 (16.7)	8 (23.5)	0.71	0.4
Left-hemisphere stroke, *n* (%)	27 (56.3)	20 (58.8)	0.05	0.817

Data are presented as mean ± SD, median (IQR), or number (%). ICA, internal carotid artery; MCA, middle cerebral artery; IVT, intravenous thrombolysis; EVT, endovascular thrombectomy; mTICI, modified Thrombolysis in Cerebral Infarction; TOAST, Trial of ORG 10172 in Acute Stroke Treatment. Successful recanalization was defined as mTICI grade 2b or 3. An asterisk denotes statistical significance at *p < 0.05.

### 3.2 Quantitative perfusion disparities

**Figure 1:**
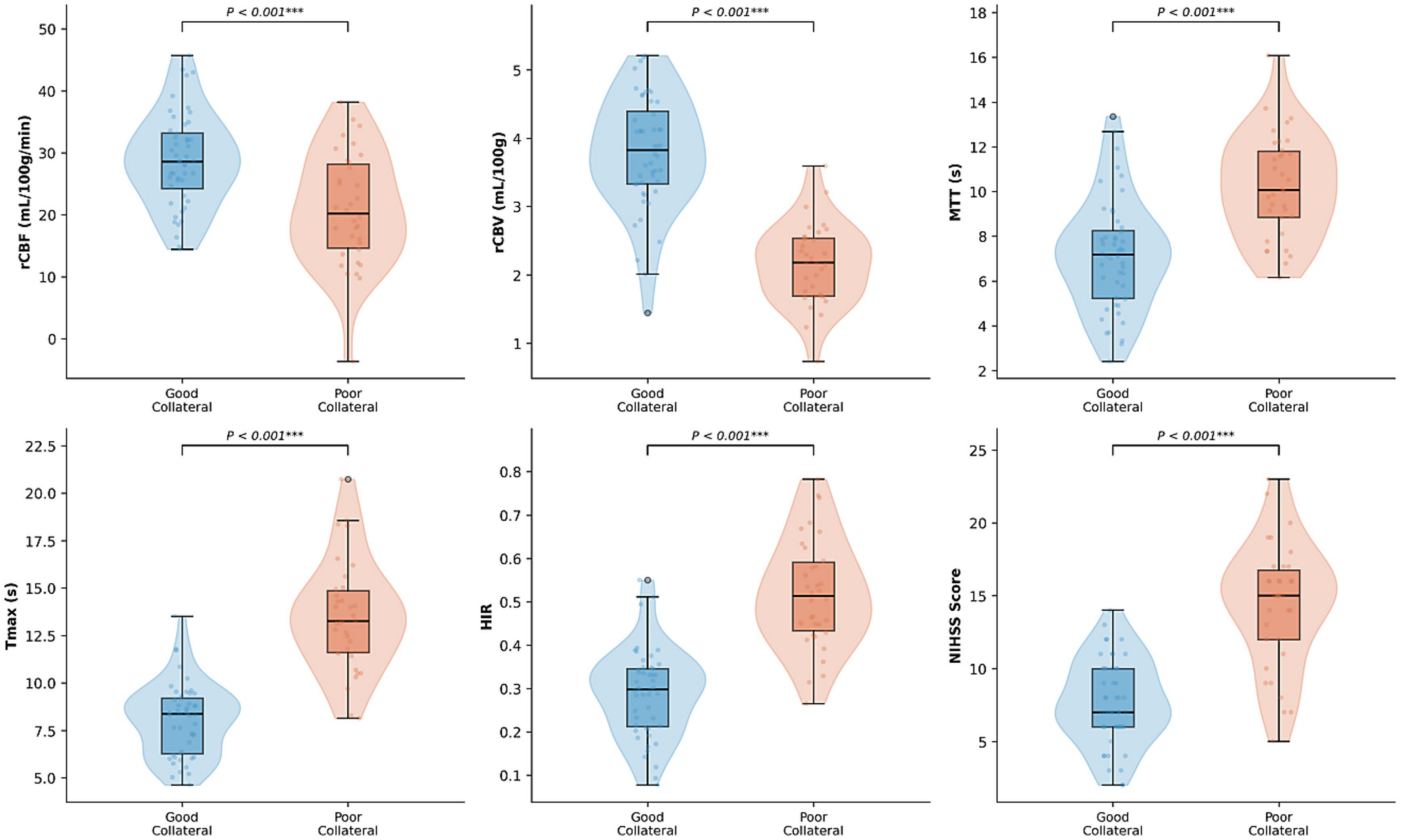
Violin and box plots illustrating the distribution of quantitative CTP parameters stratified by collateral circulation status. Overlaid box plots denote medians and interquartile ranges, alongside individual data points for each parameter. All intergroup differences achieved high statistical significance (*p* < 0.001).

Hemodynamic metrics captured by CTP clearly delineated the two populations (Figure 1). Patients blessed with robust collateral networks registered far higher cerebral perfusion benchmarks. Their rCBF (30.5 ± 8.2 vs. 21.6 ± 7.8 mL/100 g/min) and rCBV (3.8 ± 0.9 vs. 2.1 ± 0.7 mL/100 g) volumes eclipsed those of the poor collateral cohort (*p* < 0.001 for both).

Conversely, the temporal delay indices favored the good collateral group. Both MTT (7.2 ± 2.5 vs. 9.8 ± 3.0 s) and Tmax (7.8 ± 2.1 vs. 13.2 ± 3.5 s) were markedly truncated. Most strikingly, the HIR plummeted in the well-collateralized patients (0.28 ± 0.10 compared to 0.52 ± 0.12, *p* < 0.001). Volumetrically, the protective effect was undeniable. The robust cohort suffered a fraction of the ischemic core volume (15.6 ± 12.8 vs. 42.3 ± 28.5 mL, *p* < 0.001) while preserving a vastly expanded salvageable penumbra (85.2 ± 35.6 vs. 52.8 ± 28.3 mL, *p* < 0.001) (Table 2).

**Table 2:** Hemodynamic divergence based on collateral network status.

Hemodynamic metric	Robust collaterals (*n* = 48)	Poor collaterals (*n* = 34)	t/Z-statistic	*P* value
rCBF (mL/100 g/min)	30.5 ± 8.2	21.6 ± 7.8	4.98	< 0.001
rCBV (mL/100 g)	3.8 ± 0.9	2.1 ± 0.7	9.32	< 0.001
MTT (seconds)	7.2 ± 2.5	9.8 ± 3.0	−4.29	< 0.001
Tmax (seconds)	7.8 ± 2.1	13.2 ± 3.5	−8.67	< 0.001
HIR	0.28 ± 0.10	0.52 ± 0.12	−10.05	< 0.001
Ischemic core (mL)	15.6 ± 12.8	42.3 ± 28.5	−5.52	< 0.001
Ischemic penumbra (mL)	85.2 ± 35.6	52.8 ± 28.3	4.46	< 0.001
Mismatch ratio	6.8 ± 4.2	2.5 ± 1.8	5.74	< 0.001

Data represented as mean ± SD.

### 3.3 Modeling collateral deficits via CTP biomarkers

**Figure 2:**
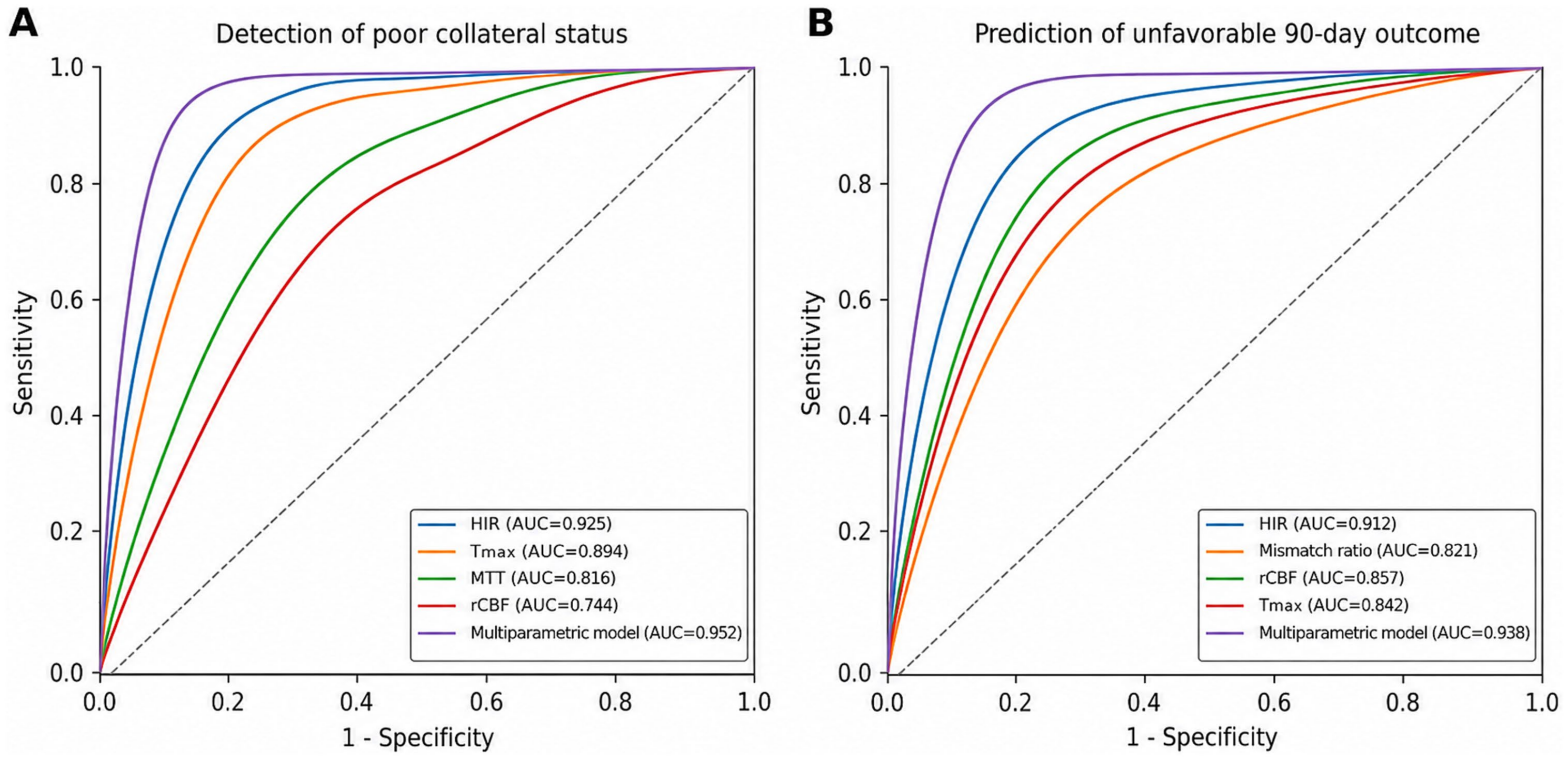
Receiver operating characteristic (ROC) curves evaluating the prognostic capacity for **(A)** collateral circulation viability and **(B)** short-term functional recovery. Panel **A** maps the discriminative strength of isolated and multiparametric CTP indices in detecting poor collateral status. Panel **B** depicts the predictive performance of HIR, mismatch ratio, rCBF, Tmax, and the multiparametric model for unfavorable 90-day functional outcome, defined as mRS 3–6. The AUCs of HIR and mismatch ratio were compared using the DeLong test. The dashed diagonal reference line indicates theoretical chance performance (AUC = 0.5).

To isolate the predictive accuracy of these perfusion metrics regarding poor collateralization, we executed ROC curve analytics (Figure 2). The results firmly established HIR as the preeminent solitary biomarker. Generating an AUC of 0.925 (95% CI: 0.865–0.962), a diagnostic cutoff of 0.40 yielded 87.5% specificity and 82.4% sensitivity. Tmax followed closely, capturing an AUC of 0.894 (95% CI: 0.825–0.941) at a 10.5-s threshold. Predictive utility tapered slightly for MTT (AUC 0.816) and rCBF (AUC 0.744).

What emerges from this analysis is the undeniable power of data integration. By fusing HIR, Tmax, and rCBF into a single predictive matrix, the combined model achieved a stellar AUC of 0.952 (95% CI: 0.901–0.982). This multiparametric triad thoroughly eclipsed rCBF evaluated in isolation (*p* = 0.003, DeLong test) and held a marginal, yet statistically significant, edge over HIR alone (*p* = 0.048) (Table 3).

**Table 3:** ROC analytics for detecting impoverished collateral networks.

Predictive marker	Computed AUC (95% CI)	Diagnostic threshold	Sensitivity (%)	Specificity (%)	*P* value
HIR	0.925 (0.865–0.962)	0.40	82.4	87.5	< 0.001
Tmax	0.894 (0.825–0.941)	10.5 s	79.4	85.4	< 0.001
MTT	0.816 (0.735–0.882)	8.2 s	73.5	77.1	< 0.001
rCBF	0.744 (0.656–0.821)	25.3	70.6	72.9	< 0.001
Multiparametric model	0.952 (0.901–0.982)	—	88.2	91.7	< 0.001

The multiparametric model represents the fusion of HIR, Tmax, and rCBF.

### 3.4 Correlating perfusion with clinical reality

**Figure 3:**
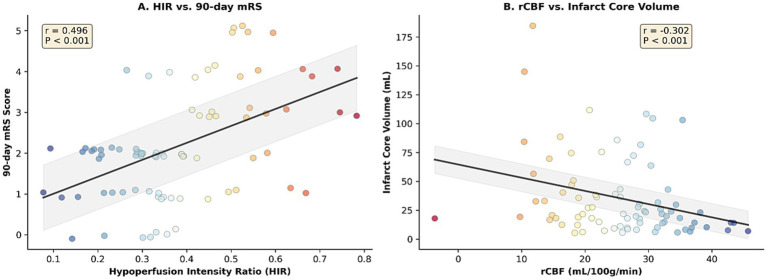
Bivariate correlational analysis bridging acute CTP hemodynamics and ultimate clinical endpoints. **(A)** Scatter plot demonstrating the robust positive correlation mapping baseline HIR against 90-day mRS severity (*r* = 0.496, *p* < 0.001). **(B)** Scatter plot outlining the inverse scaling between rCBF and final ischemic core expansion (*r* = −0.302, *p* = 0.006). Solid trend lines represent the linear regression fit, bounded by shaded regions denoting the 95% confidence intervals.

Evaluating the raw correlational data (Figure 3) reveals tight linkages between early hemodynamic signatures and long-term functional recovery. A highly significant, moderate positive scaling was observed between HIR and the ultimate 90-day mRS score (r = 0.496, *p* < 0.001; Figure 3A). Simply put, as the perfusion delay ratio expanded, patient outcomes deteriorated. Tmax mirrored this destructive trajectory (r = 0.453, *p* < 0.001).

Predictably, flow volume behaved inversely. Depressed rCBF strongly tracked with catastrophic clinical outcomes (r = −0.385, *p* < 0.001). Further validating this mechanism, lower regional perfusion highly correlated with massive final ischemic core volumes (r = −0.302, *p* = 0.006; Figure 3B).

### 3.5 Projecting 90-day functional outcome trajectories

Within our 82-patient cohort, 38 individuals (46.3%) achieved favorable functional outcomes (mRS 0–2), whereas 44 patients (53.7%) had unfavorable outcomes (mRS 3–6). Table 4 summarizes the quantitative CTP parameters and volumetric measurements stratified by 90-day outcome. Patients with unfavorable outcomes demonstrated significantly higher HIR and ischemic core volumes, lower mismatch ratios, depressed rCBF/rCBV, and prolonged MTT/Tmax compared with those with favorable outcomes (all *p* < 0.001), confirming the predictive value of baseline perfusion metrics for short-term prognosis.

**Table 4:** Comparison of quantitative CT perfusion parameters according to 90-Day functional outcome.

Clinical/CTP metric	Favorable outcome (mRS 0–2, *n* = 38)	Unfavorable outcome (mRS 3–6, *n* = 44)	t/Z	*P* value
HIR	0.30 ± 0.12	0.50 ± 0.13	−7.21	<0.001*
Mismatch ratio	6.2 ± 3.9	3.1 ± 2.0	5.68	<0.001*
Ischemic core (mL)	17.5 ± 13.0	40.2 ± 27.5	−5.13	<0.001*
Ischemic penumbra (mL)	82.0 ± 36.0	55.0 ± 28.0	4.21	<0.001*
rCBF (mL/100 g/min)	29.8 ± 8.0	21.5 ± 7.5	5.12	<0.001*
rCBV (mL/100 g)	3.7 ± 0.9	2.2 ± 0.7	7.21	<0.001*
MTT (s)	7.5 ± 2.6	9.8 ± 3.1	−4.10	<0.001*
Tmax (s)	8.0 ± 2.3	13.0 ± 3.4	−7.80	<0.001*

Mismatch-ratio comparisons were based on 80 patients because two patients with an ischemic core volume of 0 mL had an undefined mismatch ratio.

ROC analysis demonstrated that HIR was the strongest individual CTP predictor of unfavorable 90-day functional outcome, with an AUC of 0.912 (95% CI: 0.848–0.955). At an optimal cutoff value of 0.40, HIR yielded a sensitivity of 84.1% and a specificity of 84.2%. Mismatch ratio demonstrated good prognostic discrimination, with an AUC of 0.821 (95% CI: 0.721–0.895) in 80 patients. A mismatch ratio cutoff of ≤4.05 predicted unfavorable 90-day outcome with a sensitivity of 79.5% and specificity of 76.3%. Direct comparison using the DeLong test in the same patient subset showed that the AUC of HIR was significantly greater than that of mismatch ratio (AUC difference = 0.091, 95% CI: 0.009–0.173; Z = 2.17, *p* = 0.030). The rCBF and Tmax parameters yielded AUCs of 0.857 and 0.842, respectively (Figure 2).

The combined model incorporating HIR, rCBF, and Tmax showed the highest prognostic discrimination, with an AUC of 0.938 (95% CI: 0.882–0.971), sensitivity of 86.4%, and specificity of 89.5% (Table 5). Patients with unfavorable outcomes exhibited more severe initial neurological deficits and greater hypoperfusion at baseline.

**Table 5:** ROC performance of CT perfusion parameters for predicting unfavorable 90-Day functional outcome.

Predictive marker	AUC (95% CI)	Optimal cutoff	Sensitivity (%)	Specificity (%)	*P* value
HIR	0.912 (0.848–0.955)	>0.40	84.1	84.2	<0.001
Mismatch ratio*	0.821 (0.721–0.895)	≤4.05	79.5	76.3	<0.001
rCBF (mL/100 g/min)	0.857 (0.770–0.918)	≤25.6	81.8	78.9	<0.001
Tmax (s)	0.842 (0.752–0.907)	>10.3	79.5	81.6	<0.001
Multiparametric model†	0.938 (0.882–0.971)	—	86.4	89.5	<0.001

The multiparametric model included HIR, rCBF, and Tmax. Mismatch-ratio ROC analysis included 80 patients because two patients had an ischemic core volume of 0 mL. The AUC of HIR was significantly greater than that of mismatch ratio according to the DeLong test (AUC difference = 0.091, Z = 2.17, *P* = 0.030). HIR = hypoperfusion intensity ratio; AUC = area under the receiver operating characteristic curve.

In the multivariable logistic regression model for unfavorable 90-day functional outcome, HIR remained independently associated with outcome after adjustment for age, sex, admission NIHSS, ischemic core volume, IVT, EVT, successful reperfusion, TOAST classification, atrial fibrillation, and onset-to-imaging time. Each 0.1-unit increase in HIR was associated with a 36% increase in the odds of unfavorable outcome (adjusted OR = 1.36, 95% CI: 1.10–1.68, *p* = 0.004). Admission NIHSS (adjusted OR = 1.10 per point, 95% CI: 1.02–1.19, *p* = 0.007) and ischemic core volume (adjusted OR = 1.03 per mL, 95% CI: 1.01–1.05, *p* = 0.020) also remained independently associated with unfavorable outcome. In the parsimonious primary model, HIR, admission NIHSS, and ischemic core volume remained independently associated with unfavorable 90-day functional outcome, whereas IVT, EVT, successful reperfusion, and TOAST classification did not reach statistical significance. No important multicollinearity was detected among variables retained in the final model, with all variance inflation factors below 2.5. The Hosmer–Lemeshow test indicated acceptable model calibration (χ^2^ = 6.21, *p* = 0.624) (Table 6).

**Table 6:** Parsimonious multivariable logistic regression model for unfavorable 90-Day functional outcome.

Variable	β	SE	Adjusted OR	95% CI	*P* value
Admission NIHSS, per point	0.104	0.037	1.11	1.03–1.20	0.005*
Ischemic core volume, per mL	0.029	0.012	1.03	1.01–1.05	0.018*
HIR, per 0.1-unit increase	0.322	0.108	1.38	1.12–1.70	0.003*
IVT	−0.186	0.487	0.83	0.32–2.16	0.703
EVT	0.218	0.549	1.24	0.42–3.65	0.691
Successful reperfusion†	−0.684	0.431	0.5	0.22–1.17	0.112
Cardioembolic TOAST subtype‡	0.438	0.468	1.55	0.62–3.88	0.349

The primary model was restricted to seven clinically relevant predictors to reduce overfitting. HIR was analyzed per 0.1-unit increase. †Successful reperfusion was defined as mTICI 2b–3 among EVT-treated patients. ‡The cardioembolic subtype was compared with all non-cardioembolic TOAST subtypes. Atrial fibrillation was not entered simultaneously with the cardioembolic TOAST subtype because of collinearity and was evaluated in a separate sensitivity analysis. All variance inflation factors were <2.5. The Hosmer–Lemeshow goodness-of-fit test showed acceptable calibration (χ^2^ = 6.21, *p* = 0.624).

In a separate multivariable logistic regression model using poor collateral status as the dependent variable, the cardioembolic TOAST subtype was independently associated with poor collaterals (adjusted OR = 1.85, 95% CI: 1.05–3.26, *p* = 0.033), after adjustment for age, admission NIHSS, target-vessel location, and onset-to-imaging time. This collateral-status model was analyzed separately from the 90-day outcome model.

This mathematical reality mapped perfectly onto the clinical outcome data. Nearly 80 % (79.2%) of patients with robust baseline collaterals recovered favorably. In stark contrast, merely 20.6% of the poor collateral group achieved functional independence (*p* < 0.001).

## 4 Discussion

The principal contribution of this study lies in linking two distinct levels of collateral assessment: macrovascular anatomical collateral filling visualized on multiphase CTA and downstream functional perfusion quantified by CTP. Rather than treating HIR solely as another surrogate collateral score, our analysis positions it as a tissue-level marker of the effectiveness with which visible arterial collateral pathways preserve microvascular perfusion. This distinction is clinically relevant because anatomically similar collateral patterns may not produce equivalent tissue viability. We identified an HIR threshold of 0.40 for detecting poor collateral status (AUC 0.925). After adjustment for intravenous thrombolysis, endovascular thrombectomy, successful recanalization, TOAST classification, age, sex, atrial fibrillation, and onset-to-imaging time, HIR remained independently associated with unfavorable 90-day outcome: each 0.1-unit increase raised the odds of severe disability or death by 36% (adjusted OR 1.36, 95% CI 1.10–1.68, *p* = 0.004). Moreover, HIR provided significantly greater prognostic discrimination than mismatch ratio in the present cohort (AUC 0.912 vs. 0.821, DeLong *p* = 0.030), and a combined CTP model incorporating HIR, Tmax, and rCBF achieved an outstanding AUC of 0.938. These findings extend the conventional collateral–perfusion–outcome relationship by quantitatively characterizing how macrovascular collateral anatomy is translated into functional tissue preservation, and they advocate for a multifaceted, integrated approach to hemodynamic assessment in the emergency setting.

The physiological necessity of collateral flow is undisputed. By buffering the rapid decay of ischemic penumbra into dead core tissue, these networks heavily dictate the ultimate success of reperfusion therapies (3, 4). Historically, mapping these vessels required digital subtraction angiography or single-phase CTA, both suffering from invasive risks, operator subjectivity, and poor temporal resolution (21). CTP bypasses these hurdles by offering dynamic, tissue-level hemodynamic surveillance. In our cohort, well-collateralized patients exhibited shorter MTT and Tmax, higher rCBF and rCBV, smaller ischemic cores, and larger mismatch ratios, consistent with the physiological role of collaterals in sustaining residual tissue perfusion (22, 23). These findings appear to differ from the early multiphase CTA study by Menon et al. (18), in which collateral assessment showed stronger prognostic performance than the CTP measures available at that time. Several factors explain this apparent discrepancy. First, perfusion post-processing has evolved substantially since 2015; contemporary delay-insensitive algorithms, standardized rCBF <30% core thresholds, Tmax >6 s hypoperfusion definitions, and derived metrics such as HIR provide a more robust representation of tissue-level hemodynamic impairment. Second, differences in cohort composition—sample size, distribution of intracranial ICA, M1, and M2 lesions, baseline core volume, treatment-selection criteria, and recanalization rates—may have influenced the results. Thus, our findings should not be interpreted as contradicting the prognostic importance of mCTA collaterals; rather, they suggest that modern CTP metrics provide complementary functional information downstream from the anatomical collateral pathways visualized on CTA.

Among individual CTP parameters, HIR showed the highest discriminative performance, with an AUC of 0.925 at a 0.40 threshold for detecting poor collaterals. This reinforces a growing scientific consensus elevating HIR to a premier imaging biomarker. Previously, Niu et al. (11) mapped HIR against CTA-derived collateral metrics in large vessel occlusions, finding an AUC of 0.86 at a 0.45 threshold. Evaluating anterior circulation pathologies using RAPID software, Potreck et al. (24) noted that relative CBF narrowly beat HIR in their specific cohort. The slight threshold variance we observed (0.40 vs. 0.45) is likely a benign byproduct of differing post-processing algorithms and nuanced cohort compositions. The underlying pathophysiology of HIR is highly intuitive. By measuring the volume of catastrophic perfusion failure (Tmax >10 s) against total hypoperfusion (Tmax >6 s), HIR quantifies the depth of the ischemic crisis (25). When strong collaterals reroute blood, transit delays are mitigated, and the fraction of tissue suffering severe delay shrinks, dragging HIR down. Conversely, failing collaterals plunge massive swaths of tissue into severe, prolonged ischemia, rocketing HIR upward (12, 14). Software-assisted calculation standardizes HIR derivation and substantially reduces the subjective variability associated with purely visual collateral assessment.

In the present outcome-based analysis, HIR demonstrated significantly greater prognostic discrimination than the mismatch ratio. This difference reflects the distinct biological information captured by the two indices. Mismatch ratio primarily describes the relative balance between the total hypoperfused territory (Tmax >6 s) and the established ischemic core (rCBF <30%), indicating the volume of salvageable tissue. In contrast, HIR quantifies the proportion of severely delayed tissue (Tmax >10 s) within the entire hypoperfused region, making it more sensitive to the efficiency of tissue-level collateral perfusion and the severity of microvascular transit delay, even among patients with apparently similar mismatch profiles. No solitary metric can fully describe an event as complex as cerebral ischemia. Where rCBF maps the physical absence of blood, and Tmax outlines the broader spatial footprint of the delay, HIR quantifies the actual efficiency of the biological rescue networks (9, 10). Combining these three parameters (HIR, Tmax, and rCBF) pushed our predictive AUC to 0.938, providing clinicians with a three-dimensional view of the ischemic battlefield.

Beyond mere physiological mapping, these CTP numbers directly forecast clinical realities. The robust positive correlation between baseline HIR and 90-day mRS (r = 0.496, *p* < 0.001) is clinically actionable. After adjustment for age, sex, admission NIHSS, ischemic core volume, IVT, EVT, successful reperfusion, TOAST classification, atrial fibrillation, and onset-to-imaging time, HIR remained independently associated with unfavorable 90-day outcome, suggesting that its prognostic information is not fully explained by baseline neurological severity, treatment selection, reperfusion success, or stroke etiology. This aligns seamlessly with Sun et al.’s research establishing CBV indices as late-window functional predictors (26) and with a large multicenter analysis by Salim et al. that cemented high HIR values as a primary driver of catastrophic outcomes in large-core stroke populations (27). Patients with poor initial perfusion parameters face a grim trajectory. Armed with this knowledge early, clinicians can pivot their management strategies: these high-risk patients may necessitate more aggressive interventions, hyper-vigilant ICU monitoring, or the immediate deployment of novel neuroprotective agents (28). We also observed a significantly elevated prevalence of atrial fibrillation and cardioembolic (CE) strokes in the poor-collateral cohort. In a separate model focusing on collateral status, the cardioembolic TOAST subtype was independently associated with poor collaterals (adjusted OR 1.85, 95% CI 1.05–3.26, *p* = 0.033). This association likely reflects the abrupt occlusion caused by relatively large cardiac emboli, which provides limited time for ischemic preconditioning or collateral recruitment (29). This pathophysiology underlies the poorer collateral compensation observed in CE patients, highlighting the importance of etiology-aware prognostication.

We acknowledge several limitations. Primarily, the retrospective design introduces inherent selection biases—notably the exclusion of patients lacking adequate CTP scans. Second, the relatively small sample size of 82 limits statistical power for detailed subgroup analyses and the generalizability of our findings. All CTP datasets were processed using a single semiautomated platform, CT Perfusion 4D (30). Fully automated platforms such as RapidAI are increasingly employed in international multicenter trials, but CT Perfusion 4D remains routinely available in many hospitals where automated platforms have not been universally implemented due to cost, licensing, infrastructure, or workflow constraints. Operator dependence was minimized through a standardized processing protocol, blinded dual-reader review, predefined thresholds, and senior adjudication, with manual intervention limited to correction of technically inadequate automatic outputs. Additionally, although multiphase CTA enabled visualization of both arterial collateral filling and downstream venous drainage, venous outflow parameters were not systematically quantified. Emerging venous biomarkers, including cortical vein opacification scores and composite arterial–venous models such as COVES or PRECISE-type assessments, may provide further information on microvascular patency and tissue-level collateral efficiency. Our analysis was designed primarily to examine the relationship between arterial collateral status and tissue-level perfusion parameters; therefore, venous outflow was outside the prespecified analytical framework. Future prospective studies should integrate arterial collateral grades, quantitative venous outflow indices, and CTP biomarkers into a unified multivariable model to determine whether combined arterial–venous assessment further improves prognostic accuracy. Finally, because different software packages use different deconvolution algorithms and post-processing definitions, the cutoff values reported here should not be assumed to be directly interchangeable with those generated by RapidAI or other platforms and require external cross-platform validation.

Synthesizing these findings, it is clear that multi-slice CTP quantitative parameters—dominated by HIR and Tmax—are highly formidable tools. They seamlessly identify collateral network failure while simultaneously predicting 90-day functional disability in acute ischemic stroke patients. Integrating these distinct hemodynamic markers significantly amplifies prognostic power. By adopting multiparametric CTP analysis into standard emergency protocols, neurologists can dramatically sharpen their clinical decision-making, offering highly individualized triage and precision therapeutic targeting in the critical early hours of stroke management.

## Data Availability

The original contributions presented in the study are included in the article/supplementary material, further inquiries can be directed to the corresponding author.
